# Z-DNA and Z-RNA in human disease

**DOI:** 10.1038/s42003-018-0237-x

**Published:** 2019-01-07

**Authors:** Alan Herbert

**Affiliations:** Discovery, InsideOutBio, 42, 8th Street, Unit 3412, Charlestown, MA 02129 USA

## Abstract

Left-handed Z-DNA/Z-RNA is bound with high affinity by the Zα domain protein family that includes ADAR (a double-stranded RNA editing enzyme), ZBP1 and viral orthologs regulating innate immunity. Loss-of-function mutations in ADAR p150 allow persistent activation of the interferon system by Alu dsRNAs and are causal for Aicardi-Goutières Syndrome. Heterodimers of ADAR and DICER1 regulate the switch from RNA- to protein-centric immunity. Loss of DICER1 function produces age-related macular degeneration, a different type of Alu-mediated disease. The overlap of Z-forming sites with those for the signal recognition particle likely limits invasion of primate genomes by Alu retrotransposons.

## Abstract (web summary)

Alan Herbert discusses the properties of Z-DNA and Z-RNA, interactions with ADAR and other Z-binding proteins, and the role these elements play in disease. He also discusses the implication of Z-forming sites in genome evolution.

Z-DNA is the left-handed conformer of double-stranded DNA that normally exists in the right-handed Watson-Crick B-form. The flip from the B-form to the Z-form occurs when processive enzymes such as polymerases and helicases generate underwound DNA in their wake. The existence of Z-DNA was unexpected and its discovery accidental, the structure trapped in the first synthetic DNA ever crystallized. Initially the biological importance of Z-DNA was overestimated, after which it has been underappreciated (“We tend to overestimate the effect of a technology in the short run and underestimate the effect in the long run” - Roy Anara). An important inflection point has been the identification of the Z-DNA binding domain named Zα^1,2^ from the dsRNA editing protein ADAR^3,4^. This domain’s specificity for the left-handed conformation of Z-DNA was shown in a series of high-resolution NMR and X-ray studies^5,6^. The interactions between Zα and Z-DNA are conformation-specific, with no base-specific contacts.

Over the past 25 years, work by many outstanding scientists has established a clearer view of the roles played by ADAR, along with other Z-binding proteins, in the interface between the RNA and protein worlds in health and disease. Central to these findings were genetic studies revealing an essential role for the interferon-induced p150 isoform of ADAR that includes the Zα domain in the negative regulation of immune responses induced by dsRNA. In addition, Zα was found to bind the left-handed Z-RNA conformation of dsRNA without sequence specificity, providing a mechanism for targeting ADAR to dsRNA editing substrates independently of the three ADAR dsRNA binding domains that recognize the more common right-handed A-form dsRNA conformation. Mutations that reduce p150 Z-binding along with those that impair enzymatic activity cause interferonopathies such as Aicardi-Goutières Syndrome. The dsRNAs that induce disease arise from transcripts with inverted repeats, which fold back and base pair with each other. Most commonly, these dsRNAs derive from Alu retroelements but they are also generated during viral infections.

The Z-conformation, both as Z-DNA and Z-RNA, has likely played an essential role in limiting Alu retroelement invasion of primate genomes during evolution. Besides ADAR, key partners in this battle have been the signal recognition particle proteins SRP9 and SRP14 (on which Alu retrotransposition depends) and DICER1 (an endoribonuclease that heterodimerizes with ADAR and initiates RNA interference against single copy Alu transcripts, which are unlikely to form the long dsRNA required either for editing by ADAR or for the formation of Z-RNA). SRP9/14 dimers bind to Alu sequences capable of Z-formation, with stronger Z-formers found in those elements most successful at invading the genome. Indeed, loss of Z-forming potential is associated with loss of SRP9/14 binding and diminished Alu invasion. Recognition of Z-formation by the Zα domain targets Alu dsRNA for editing by ADAR, while recognition of Z-DNA by ADAR recruits DICER1 machinery to single copy genomic insertions, both enzymes serving to limit further retrotransposition. Loss of DICER1 function is associated with the accumulation of Alumers and inflammasome activation, leading to age-related macular degeneration (AMRD). The need to defend against retroelements provides a rationale for maintaining Z-forming segments in the genome. Other roles for the left-handed conformation in the readout of genomic information exist and these too show an association with disease. A genome-wide analysis reveals that both dsRNA editing and known disease genes are enriched for long Z-DNA forming segments.

In this review, I discuss the properties of Z-DNA and Z-RNA, and detail how the Zα domain of ADAR limits Alu retrotransposition and protects against human disease. I examine the impact of the Z-conformation on primate evolution, and outline key questions that remain in the field.

## Z-DNA and Zα domains

**Fig. 1:**
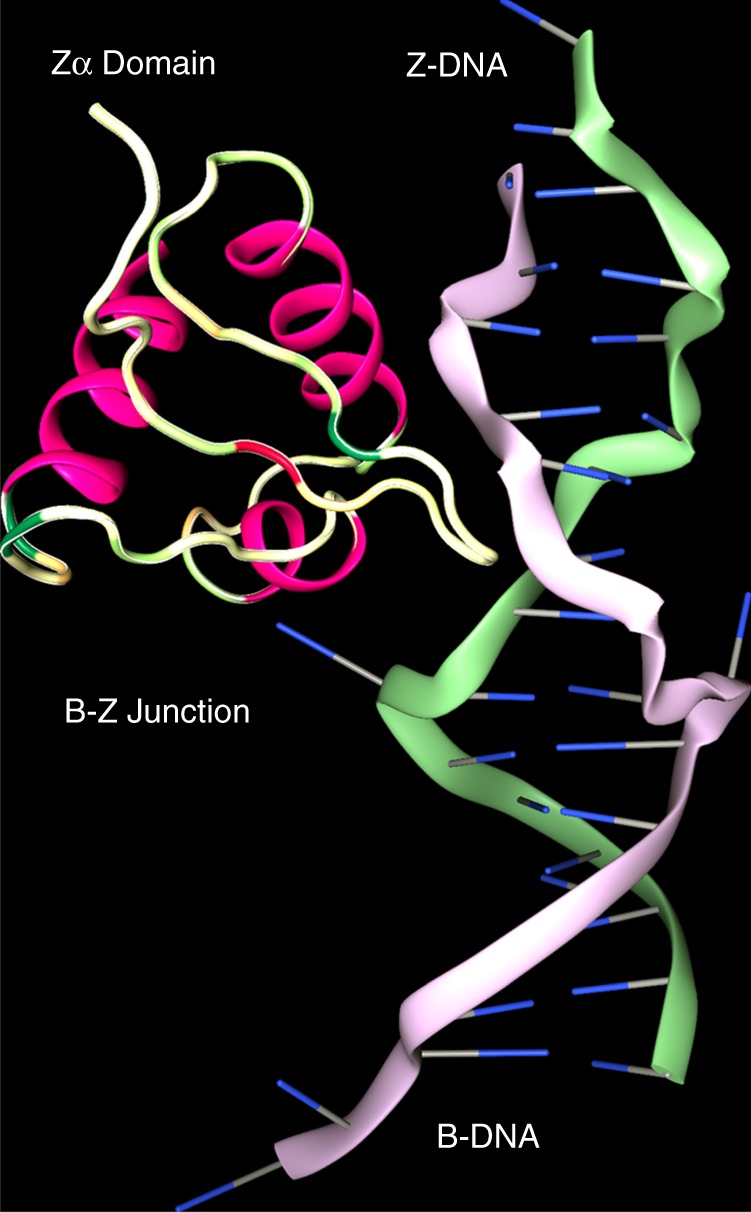
The B−Z junction. Z-DNA is a conformer of B-DNA stabilized both by negative superhelical stress and by binding of the Zα domain of ADAR1. Zα is conformation specific, contacting the DNA backbone through the α3 helix and its carboxy-terminal β Hairpin but not making any sequence-specific contacts with bases. Formation of the B−Z junction is driven entropically by the eversion of two bases from the helix, a process further favored when non-Watson-Crick basepairs, such as mismatches, are present at this position (PDB structure 2ACJ)

The discovery of Z-DNA occurred when an unusual DNA conformation was observed upon placing poly(dC-dG) in 5 M NaCl^7^. Its structure was revealed when the crystal of d(CG)_6_ was solved^8^. The left-handed helix was built from a dinucleotide repeat where the usual *anti* conformation of bases alternated with the unusual *syn* form (where the purine or pyrimidine base projects over the (deoxy)ribose ring, perpendicular to its plane, rather than pointing away from it as it does in *anti*), giving rise to a zig-zag backbone structure, features captured by the name Z-DNA (Fig. 1).

The demonstration that B-DNA could be flipped to form Z-DNA by negative superhelical stress without strand cleavage brought the left-handed conformation into the realm of biology^9^. The ease of Z-formation varied with sequence—d(CG)_*n*_ flips better than d(TG)_*n*_ and d(GGGC)_*n*_ with less torsion than d(TA)_*n*_—reflecting the energetic cost of pushing one base of each pair into *syn*. The major barrier to the initiation of Z-formation was the additional energy required to create two B−Z junctions^9,10^. Once nucleated, the transition from B- to Z-DNA is cooperative. Z-DNA formation could be driven by processive enzymes, such as polymerases and helicases, that leave underwound DNA in their wake.

Structural studies of the Zα domain of ADAR confirmed that it is specific to the Z-conformation without any base-specific contacts. This domain has a helix-turn-helix motif similar to that found in B-DNA binding proteins and a binding site of 6 basepairs^6,11^. Cocrystallization of the Cyprinid herpes virus 3 ORF11 Zα with d(T(CG)_9_) DNA identified a guanosine base-specific contact outside the core-binding site that may help stabilize the B−Z transformation^12^. Further structural studies, enabled by Zα, detailed B−Z and Z−Z junctions at atomic resolution. The B−Z junction extruded a basepair from the helix where the phosphate backbone reverses direction^13^ (Fig. 1) and rendered each base susceptible to damage by mutagens or to modification by enzymes^14^. The Z−Z DNA junction, where out-of-phase Z-helices meet, was found unstacked and open to intercalation^15^.

Biophysical studies have demonstrated that Zα does not induce the Z-DNA conformation but rather is recruited after formation^16^. Dissociation from Z-DNA is slow (measured in hours), a feature that most likely enabled the initial purification of this domain^2^. Further studies revealed that the Zα domain stabilizes Z-DNA formed by G:T mismatches, by triplet d(GAC)_4_ repeats with A:A mismatches^17^, and in sequences with as many as three consecutive d(CC) dinucleotide steps^18^. Zα binds to Z-RNA in a manner very similar to its interaction with Z-DNA but with differences in solvation due to the 2′-OH group of RNA^19^. Unexpectedly, formation of a Zα-complex with double-stranded nucleic acids is most rapid with DNA−RNA hybrid duplexes, reflecting the lower energetic cost of junction formation, not a higher affinity of Zα for this structure^20^. The winged-helix-turn-helix Zα motif was found in 182 other proteins (SMART Domain SM00550) representing orthologs of ADAR1, ZBP1, PKZ, E3L and ORF112 from different species. Structural studies have confirmed that many of these bind Z-DNA, including a number of viral proteins, that, like ADAR, play a role in the innate immune response and are essential for viral infectivity^12,21–26^. The related Zβ domain of human ZBP1 also binds to Z-DNA but uses a different set of contact residues^22^, suggesting that there are even more divergent members of the winged-helix-turn-helix Z-DNA binding family. Zα also binds the parallel strand G-quadruplex formed by the MYC promoter. The Zα residues engaging the G-quadruplex were shown by NMR to differ from those contacting the Z-DNA surface^27^. In a separate study, a left-handed quadruplex with a *syn-anti* dinucleotide step was described^28^. Another motif with a Z-like dinucleotide *syn-anti* conformation, called a Z-turn, was found in RNA junctions, ribosome−protein interactions^29^, the CUG splicing protein^30^ and the IFIT5 RNA protein complex^31^. So far, none of the interactions have been base-specific.

## ADAR and dsRNA

ADAR was first identified as an enzyme that deaminated adenosines in regions of dsRNA to produce inosine^32,33^. The edits made by ADAR in codons alter protein sequence since inosine is translated as guanosine, while those modifying splice sites and untranslated regions (UTR) change the isoform mix and transcript stability. Editing of miRNA dsRNA precursors also occurs and alters expression of miRNAs and the genes that they regulate^34^. Altering an A:C mismatch to an I:C basepair favors dsRNA formation, while editing of an A:U basepair to I:U is destabilizing^34^.

ADAR is expressed as two isoforms, p150 and p110. Both have a Zβ domain of unknown function, three dsRNA binding domains, and the catalytic domain^35^. Only the p150 isoform has the Zα domain. The p150 isoform is induced by interferon and is present predominantly in the cytoplasm, a localization modulated by nucleoytoplasmic shuffling^36^. p110 is constitutively produced and nuclear^37^. ADAR p110 regulates 3′ UTR stability in stress responses^38^, affecting translation efficiency^34^. The ADAR catalytic domain is fully functional without the attachment to the Zα, Zβ or dsRNA binding domains and edits hairpins with stems as short as 15 basepairs^39^. Like Zα, it also binds DNA/RNA hybrids, producing edits in both the RNA and DNA strands, a process that can lead to somatic mutation of elements within the genome and one with potential application in base-modifying therapeutics^40^.

Genetic studies revealed that ADAR deletion was embryonic lethal in mice due in part to a failure in generation of the hematopoietic system^26^. The phenotype is rescued, without any apparent developmental abnormalities, by deletion of the *IFIH1* gene that encodes the MDA5 pattern sensor for long dsRNA. MDA5 acts through the MAVS protein to initiate transcription of interferon-stimulated genes (ISGs), including p150 and type 1 interferons. Interferon amplifies the response by further increasing its own production^41^. Editing creates clusters of I:U basepairs that inhibit further MDA5 activation^42^ and destabilizes ISG transcripts, which undergo Staufen-mediated decay^34^. Critically, negative regulation of the MDA5 pathway in mice^43^, along with other interferon-induced responses^44^, is completely dependent on the ADAR p150 isoform. These findings place p150 and Zα at the eye of the interferon storm, which, left unchecked, exacerbates inflammatory diseases.

**Fig. 2:**
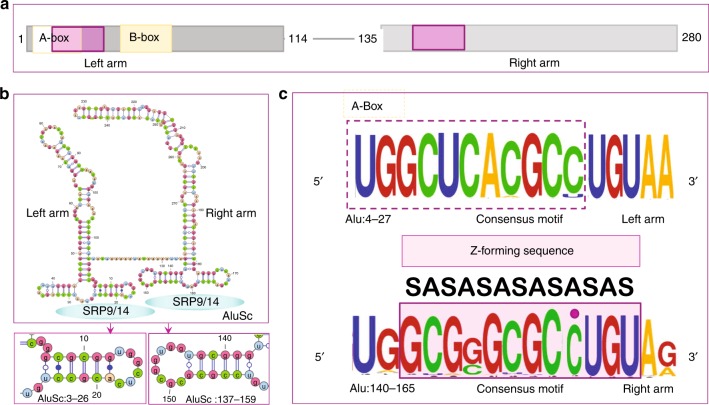
The properties of Alu repeats. **a** Alu repeats consist of a right and left arm derived originally from the 7SL RNA present in the signal recognition protein (SRP). Transcription is driven by the A and B-boxes of the left arm that are promoters for RNA polymerase III. Alu retrotransposition requires binding of the SRP9/14 heterodimer, using sites on both arms (purple box in upper panel). The site on the left arm overlaps the A-Box. **b** Each Alu arm folds independently with SRP9/14 binding sites as visualized with VARNA. **c** The left-hand SRP9/14 sequence is constrained by the interaction of the A-Box with Pol III, with very little variation apparent in the WebLogo motif for the Alu family RepeatMasker consensus sequences. The right-hand site shows more variation and has a consensus logo favoring a 6 bairpair Z-forming alternating C−G motif that can be extended to a full turn of the Z-helix by flipping the out-of-alternation cytosine residue highlighted with a purple dot. The alternating *syn-anti* (SA) of the Z-conformation is annotated

dsRNA leading to induction of interferon and p150 can arise during viral infection. Pox viral homologs of Zα such as E3L inhibit this response and are essential for viral virulence^45^. A major source of dsRNA is from endogenous retroelements, which constitute more than half the human genome^46^. These include SINEs and LINEs that are primate-specific and differ from those in mice^47^. Of these, Alu SINE sequences represent about 10% of the genome. Most Alu’s are dimeric, about 280 bases long, with a characteristic fold that has a left and right arm (Fig. 2a, b). Different Alu clades harbor distinct mutations that reflect the history and age of origin. The distribution of Alu’s within the genome is highly correlated with the density of genes^48^.

**Fig. 3:**
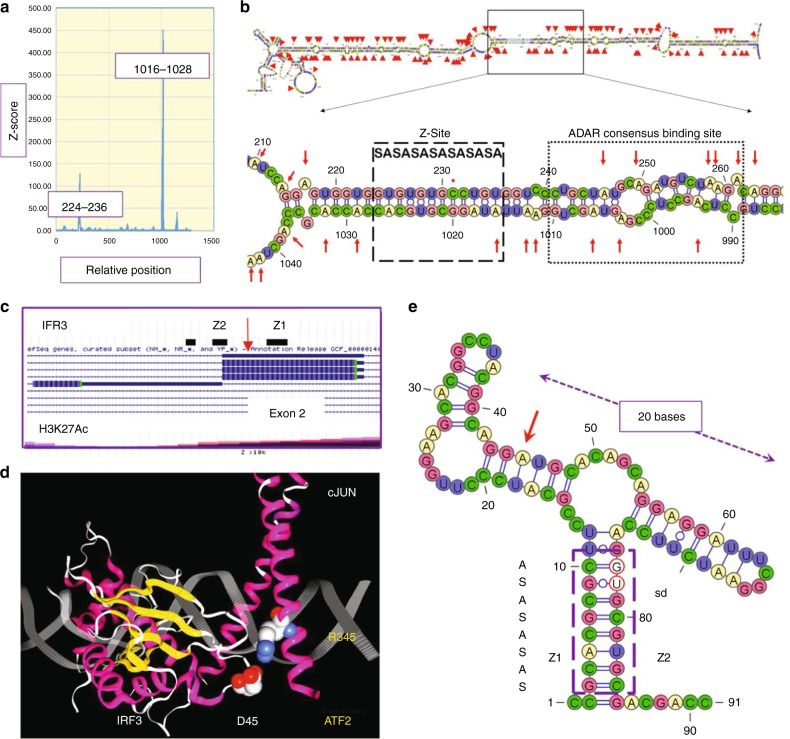
Mapping of Z-formation to editing sites. **a** The *CTSS*gene region chr1:150703475-150704757 (hg19) was analyzed for Z-DNA formation using the ZHUNT3 algorithm that generates the probability of Z-formation based on statistical mechanical calculations^10^. Z-sites map to a 5′ inverted AluSx repeat and a 3′ AluJo^57^. **b** The dsRNA foldback structure with editing sites indicated by red arrows contains an alternating *syn-anti* (SA) Z-forming segment (box with dashed lines). A dot marks the one cytosine out of alternation. The Z-site lies adjacent to the consensus ADAR binding site found by CLIP-seq (box with dotted lines)^59^. Editing sites extend 150 basepairs on either side of it. **c** Map of *IRF3* with editing site and H3K12Ac status. **d** Nonsynonymous edit of exon2 of *IRF3* changes readout of codon 45 from Glutamate (D45) to Glycine, the residue in loop 1 that hydrogen bonds to R345 of ATF2(PDB structure 1T2K). **e** A proposed minimal editing substrate. The Z-stem (box with dashes), formed by Z1 and Z2 sequences, incorporates the splice donor site (sd, bases circled in red) from exon 2. The edited A (red arrow) lies in the left arm of a 20 base long helix of similar structure to other ncRNA substrates

Alu’s are heavily edited by ADAR, commonly in regions where there are inverted repeats separated only by a few thousand bases^49–51^. Transcripts from the inverted repeats fold back on each other and pair to create long dsRNA editing substrates^52^ (Fig. 3, RADAR database^53^). Failure to clear endogenous Alu dsRNA by editing activates MDA5 and the type 1 interferon system, causing the Aicardi-Goutières Syndrome^54^. This disease is associated with mutations in ADAR p150. A recurrent causal proline to alanine mutation, P193A, lies in the Zα domain of ADAR and diminishes binding of ADAR to the Z-conformation. In vitro cell expression systems confirm that this mutation also reduces dsRNA editing^55^, confirming an essential role for Z-DNA in this disease.

Z-forming sequences are contained in Alu elements (Table 1, Fig. 2c). An example is from the cathepsin-S (CTSS) gene (Fig. 3a), which encodes a protease associated with vascular inflammation, atherosclerotic plaque rupture, and aneurysm. Deletion of the *CTSS* gene protects against vascular disease in mice but disrupts normal repair processes by reducing angiogenesis^56^. The *CTSS* gene contains an AluSx1 element 515 bases away from an AluJo element, which is in the reverse orientation. When the *CTSS* gene is transcribed, the Alu repeats fold back on each other and basepair to create a long dsRNA editing substrate for ADAR that contains a Z-RNA forming region adjacent to the consensus ADAR binding site (Fig. 3b). The single-stranded short uridine repeat sequences unmasked by editing are bound by HuR, increasing the stability and the amount of CTSS message^57^. CTSS mRNA editing is induced by interferon, which is produced during vascular inflammation, implicating binding of the Z-RNA forming region by ADAR p150 and its Zα domain in the pathogenesis of atherosclerosis.

**Table 1:** Alu family members differ in Z-DNA and editing potential

Repeat	Canonical sequence	Z-score canonical	Total annotated	Number canonical	Number edited	Fraction edited	Z-score <250	Z-score >250	Ratio (Z/Non-Z)	Mean Z-score	SE(Mean)
AluSc	gcgcgcgcctgt	5976.64	33,903	127	27,893	0.82	19,702	8191	0.42	785.07	31.31
AluSc5	gcgcgTgcctgt	1352.02	6775	145	4927	0.73	3607	1320	0.37	450.97	18.21
AluSx4	gcgcgcgcctgt	5976.64	5670	16	5164	0.91	3787	1377	0.36	748.13	53.24
AluSc8	gcAcgcgcctgt	1349.93	21,507	412	17,194	0.80	12,674	4520	0.36	747.01	37.16
AluSg	gcgcgcgcctgt	5976.64	40,784	101	36,345	0.89	26,985	9360	0.35	802.28	32.27
AluSx3	gcgcgcgcctgt	5976.64	29,020	43	24,892	0.86	19,373	5519	0.28	761.09	33.02
AluSx	gcgcgcgcctgt	5976.64	141,949	141	106,512	0.75	83,741	22,771	0.27	485.89	9.29
AluSz	gcgcgcgcctgt	5976.64	97,073	70	94,472	0.97	74,353	20,119	0.27	473.83	8.84
AluSz6	gcgcgcgcctgt	5976.64	45,181	13	41,524	0.92	34,094	7430	0.22	461.63	10.80
AluSp	gcgcATgcctgt	342.70	49,103	0	38,146	0.78	32,359	5787	0.18	433.95	12.07
AluJb	gcgcgcgcctgt	5976.64	142,591	21	117,452	0.82	101,831	15,621	0.15	480.80	8.10
AluJo	gcgcgcgcctgt	5976.64	71,274	7	53,397	0.75	47,380	6017	0.13	462.01	9.06
AluSq	gcgGgcgcctgt	257.42	21,499	1	15,103	0.70	13,403	1700	0.13	695.93	38.39
AluSq2	gcgGgcgcctgt	257.42	54,418	3	38,215	0.70	34,086	4129	0.12	448.86	12.54
AluJr	gcgcgcgcctgt	5976.64	76,315	10	59,166	0.78	52,801	6365	0.12	458.69	9.06
AluJr4	gcgcgcgcctgt	5976.64	17,432	0	14,743	0.85	13,479	1264	0.09	472.08	16.62
AluY	gcgGgcgcctgt	257.42	118,506	73	46,885	0.40	44,732	2153	0.05	469.60	15.21
AluSx1	gcgGgcgcctgt	257.42	109,158	13	73,920	0.68	72,578	1342	0.02	411.67	12.44

The results derived from a genome-wide survey of Alu elements are presented. The Z-score for each Alu RepeatMasker consensus sequence was determined using the ZHUNT3 program^10^. Differences in base sequence are capitalized. The count for each consensus sequence in hg19 (February 2009) is given along with the number of elements that actually have the consensus sequence. The number of Alu’s with edits in each family is derived from the RADAR database V2. The mean Z-score based on the actual genomic sequences for each family is calculated using sequences with a Z-Score >250. The ratio of edited Alu’s that have Z-scores >250 to those with Z-Scores <250 is given. The Pearson correlation between this ratio and the Z-score, weighted by the count of actual elements with a Z-score’s >250, is 0.69 when calculated using the wCor package

Z-DNA forming sequences in genes like *CTSS* can be predicted computationally with the ZHUNT3 program^58^. A score of 500 corresponds to a single turn of a Z-helix composed of d(AC)_6_ that adopts Z-DNA experimentally under reasonable levels of negative superhelical stress^10,58^. Higher scores imply a better Z-forming sequence and a higher likelihood of capture by Zα. In *CTSS* and many other RNAs, the Z-DNA forming elements lie within the two inverted Alu repeats (Fig. 3a). They align to form Z-RNA when the transcript folds back on itself. Editing can occur 150 bases either side of the Z-RNA region (Fig. 3b). The consensus binding site for ADAR, derived from whole-cell CHIP-seq analysis (underlined in Fig. 3b), lies adjacent to the Z-forming segment^59^ (Fig. 3b). The *CTSS* gene is just one example where structural motifs like dsRNA and Z-formation combine with sequence-specific binding proteins, like HuR, to regulate transcript levels.

Binding of Zα to Z-DNA permits localization of ADAR to transcribed Alu elements. Transfer from Z-DNA to Z-RNA can occur once the dsRNA substrate forms. The slow dissociation of Zα from Z-DNA increases time for mRNA folding by providing a temporary barrier to the passage of the next RNA polymerase through the region^60^ and provides time to complete the editing process. The association of Zα with Z-RNA further depends on the nature of the helicase(s) involved in straightening out local RNA kinks that compete with the formation of an extended dsRNA editing substrate. Processive helicases will promote Z-RNA formation by generating underwound dsRNA in their wash, more so with long substrates when the ends are fixed and unable to rotate freely.

## Alu left and right arms

It is natural to ask why Alu and why Z-DNA? Alu sequences are present mostly in GC-rich regions of the genome and consist of many families initially derived from 7SL RNA, a noncoding RNA found in the signal recognition peptide (SRP). SRP has an S-domain that binds near the peptide exit tunnel of the ribosome and an Alu domain that can stall translation^61^. Retrotransposons derived from the Alu domain arose first as monomers, then as dimers. Some of them were more invasive than others (Table 1). Transposition requires the Alu elements to hijack the Line L1 copy and paste machinery. This feat is performed by the left arm of the Alu dimer^61–63^. Crystal structures reveal that the left arm inhibits ribosomal translation by filling the gap between the two ribosomal subunits, near the tRNA A-site where translation elongation factors attach^61–63^. Like the SRP protein, the fit is mediated through the SRP9/14 protein pair.

The SRP9/14 binding site in the left Alu arm overlaps with the RNA Polymerase III A-box promoter, constraining sequence variation (Fig. 2a). The right arm sequence, lacking such restrictions, shows more variation^64^. It has a potential Z-forming (CG)_4_ core that can be extended to form a 12 bp Z-helix by flipping the an out-of-alternation cytosine (Fig. 2c). The Z-DNA forming motif is maintained in the different Alu family consensus sequences despite a high mutation rate in this region (Table 1)^65,66^, hinting that there is selection for this motif in active Alu elements . Mutations that lower transposition also lower SRP9/14 binding^64^ and are expected to lower Z-formation. The right Alu arm, but not the left arm, increases translation of newly transcribed mRNA^67^; it strips SRP9/14 proteins from the preinitiation complex, preventing the reuse of existing templates^68,69^. Mutations of the right arm that diminish SRP9/14 binding also diminish effects on translation initiation. A dimeric Alu is thus able to promote translation of recently synthesized L1 mRNA and then capture of the L1 transposase. The site of binding of SRP9/14 to the small ribosome is unknown, but the highly conserved 18S RNA sequence *tgcacgcgcgc* in helix 30 (H-30) is similar to the Alu Z-forming motif of the right arm. H-30 is solvent-exposed and contacts the anti-codon loop in the ribosomal P-site both during initiation and elongation^70,71^. H-30 is bound by uS9 (Rsp16) protein, which extends through the 40S core to contact the scaffold protein RACK1 that binds many regulators of translation initiation^72^. These include eIF3d, a cap binding protein that promotes translation of an mRNA subset when the general factor eIF4E is inactivated by stress or nutrient deprivation^73^. H-30 is thus strategically placed to choreograph the reinitiation complex. The H-30 sequence is also predicted computationally to form Z-DNA. Experimental studies of E. coli ribosome showing Zα domain cross-links to H-30 (at base 1227). The binding site is close to that for uS9^74^. Whether Zα captures H-30 in a Z-conformation is an open question. The interaction of SRP9/14 with the preinitiation complex would likely be sufficient to force Alu’s to mirror sequences like H-30 to guarantee efficient retrotransposition. The sequences need not be Z-forming in the ribosome, yet an Alu sequence similar to H-30, like that in the right arm, will flip to Z under physiological conditions when transcribed.

## Alu and Z-element evolution

**Fig. 4:**
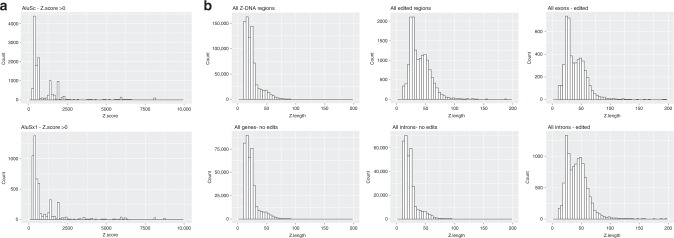
Edited genes are enriched for higher Z-scores. **a** Histogram of Z-scores less than 10,000 for AluSc annotated sequences from hg19 is right-shifted compared to AluSx1 sequences. **b** Histogram of the length of Z-elements with scores >10,000, showing enrichment of longer segments in edited genes in both exons (5′ UTR, Coding Sequences and 3′ UTR) and introns compared to nonedited genes

An overlap between SRP9/14 binding sites and Z-forming sequences is a potential weakness in the Alu retrotransposition strategy, one exploitable by ADAR to protect the host during periods of Alu retroelement invasion. Those Alu sequences with high affinity for SRP9/14 become targets for Zα and substrates for ADAR editing when they induce formation of dsRNA substrates. Persistence of the Z-DNA binding domain in ADAR1 diminishes the presence of active Alu elements. Within the primate lineage, Alu elements and ADAR evolved in tandem, one dependent upon the other. As Alu sequences mutate, they lose their ability to form Z-DNA, bind SRP9/14, regulate translation, and finally, their power to transpose, leading to their silencing as invasive agents. As seen in Table 1, the taming of Alu transposition has been very effective and left few exact matches for the proposed Z-forming consensus sequence in the Alu right arm. A trace of this history does still remain (Table 1). The fraction of each Alu family that is associated with editing varies. Of those edited, the majority form Z-DNA very poorly (Z-score < 250), suggesting that they are preferred substrates for ADAR p110 rather than p150. For those Alu’s with a Z-score > 250, the total number with edits increases with the mean Z-score for each family, the Z-score histogram of the best (AluSx) family being right-shifted compared to the worst (AluSx1) (Fig. 4a, Supplementary Data 1). The Pearson correlation, weighted by count, between editing ratio (Z-score > 250/Z-score < 250) and mean Z-score is 0.69. The Z-scores observed are consistent with transient Z-formation under physiological conditions, enabling a pause and scan mechanism where transcription is halted long enough for dsRNA editing substrates to form.

While Alu’s provide a challenge to the host, their spread generates genetic diversity and empowers natural selection^47^. Variability is further expanded by editing and other mutational mechanisms. Sequence constraints are lessened when transcription is driven by RNA Pol II rather than RNA Pol III promoters. Many examples exist where Alu sequences have been coopted to create new forms of gene regulation and novel combinations of features^75^. Added to these outcomes is the spread of Z-DNA elements that assume roles in transcription^76^, chromatin remodeling^77,78^ and in recombination^14^. When Alu elements cluster, new regulatory mechanisms evolve, such as those proposed for alternative splicing when 5′ and 3′ spice sites are brought together by pairing of Alu’s on either side of an exon. Splicing then excludes the exon from the transcript, yielding an RNA circle with no free ends. Circles are very stable and act as sponges to bind noncoding RNAs and proteins, making them unavailable for regulation of gene expression^79^. In contrast, editing by ADAR of the Alu stems allows splicing of trapped exons into other transcripts. Editing of isoforms can be selective. For example, the *SRP9* gene produces two RNA isoforms. Nonsynonymous edits are only present in isoform 2, which cannot form an Alu SRP because it lacks the residues in helix 2 to bind SRP14. SRP9 has Alu Z-elements that can time when edits occur. Z-dependent editing will be greatest after p150 is induced by interferon. The switch to isoform 2, most likely Z-dependent, will prevent Alu transposition events by downregulating SRP9.

Alternative splicing and Z-formation is enriched in genes with Alu repeats and dsRNA editing of transcripts (Supplementary Data 1, 2, 3 and below). One example is the KRAB-domain family of transcriptional repressors that expanded in response to endogenous retroviruses^80^. Editing generates additional diversity to protect against novel invaders faster than protein evolution. It is of direct selective benefit for the host.

Not all edits are associated with Alu repeats. Certainly, editing can be performed independently of either the dsRNA or Z-domains^39^. An example of a minimal Z-dependent editing domain where the substrate binding is enhanced by the Zα domain with no involvement of dsRNA binding domains may be provided by exon 2 of *IRF3*, where a nonsynonymous edit of codon 45 (Aspartic Acid → Glycine) alters a loop 1 contact of IRF3 with ATF2, the consequences of which are unknown (Fig. 3c). The foldback dsRNA has a potential 10 bp Z-RNA stem that also incorporates the exon 2 splice donor site. The alternative *IRF3* (*IRF3a*) transcript is initiated downstream from this element. The protein product lacks key residues for DNA binding and so acts as a negative modulator of IRF3 dimers, enabling fine-tuning of the interferon response^81^. A similar clustering of Z-stems overlapping splice sites in 5′ exons is present in *IRF7* and *EIF5A* genes. Binding of p150 to *EIF5A* RNA is supported by RIP-seq studies^82^.

## Long Z-elements

During evolution, some Z-forming segments have expanded, particularly d(G−T)_*n*_:d(C−A)_*n*_ repeats. Those with Z-scores greater than 10,000 are enriched in edited genes, both in exons and introns (Fig. 4 and Supplementary Data 2). Edited transcripts with genes with high Z-scores also show enrichment for alternative spicing. Around 70% of edited 3′ UTRs from genes with Z-scores greater than 10,000 are annotated as alternatively spliced compared with 60% of edited 3′ UTRs from genes with Z-scores less than 10,000. The frequencies for 5′ UTRs of edited transcripts are 66% for genes with Z-scores greater than 10,000 and 60% for genes with Z-scores less than 10,000.

d(G−T)_*n*_ repeats on the sense strand are favored by a margin of 2:1, raising the question of whether dsRNA foldback structures with wobble G:U basepairs are also bound by Zα. These long Z-regions, like Alu elements, likely have evolved many different functions beyond localization of epigenetic and DNA repair machinery^77,78^. One role may be to stall RNA polymerases and block the read-through transcription induced by viruses such as HSV that disrupt normal termination signals^83^. Stalling may also provide time for splice sites far apart to be transcribed and paired before the next polymerase enters the region. Stalling at Z-elements may optimize transcription of genes with overlapping reading frames, ensuing that each can be readout without interference from the other. Long Z-elements may block the use of an upstream transcription start site or a downstream termination signal, favoring a subset of transcripts. An example is provided by the interferon* IFNAR2* receptor gene where a long Z-element is associated with editing of intron 2. The Z-element placement favors transcription downstream from the start site used for the full-length receptor. The isoform produced encodes the soluble form of the receptor, one that modulates IFNβ stability and fine-tunes local interferon responses by signaling in *trans* through the interferon IFNAR1 receptor on adjacent cells^84,85^. Other genes with long Z-elements include genes in the RIGI/MDAS pathway with a role in regulation of the innate immune response (Reactome Pathway R-HSA-168928—adjusted *p* value = 0.02, Supplementary Data 3). Long-Z genes (containing segments with Z-scores > 10,000) are enriched for disease mutations (UP_KEYWORDS Disease Mutations, *p* value = 1.49 × 10^−8^), including those related to amino acid and vitamin metabolism, cancer, hypoxia, TGFβ, FGF and EGF signaling along with viral response pathways (Supplementary Data 3). These genes provide an experimental opportunity to map the variations in Z-element scores and locations to genetically defined disease phenotypes.

## From RNA to protein immunity

**Fig. 5:**
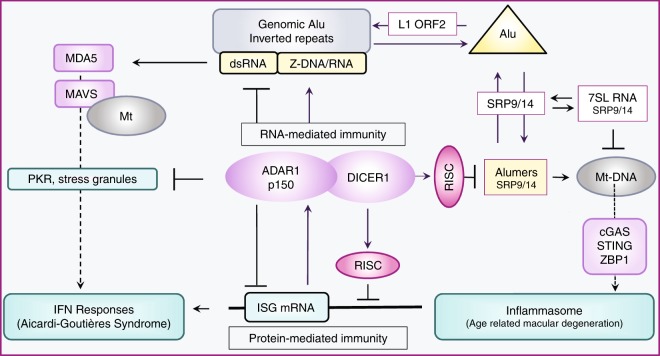
The Alu cycle and disease. The Alu cycle of retrotransposition involves the LINE1 retrotransposase (L1 ORF2), the SRP9/14 dimer and Alu dimers. Some genomic insertions result in formation of Alu inverted repeats. Transcripts from these regions fold to form dsRNA. ADAR in partnership with DICER1 regulates protein-mediated immune responses to Alu transcripts through a series of RNA-based switches. In the resting state, repression of interferon-stimulated genes (ISG) by the RISC complex is enhanced through protein−protein interactions between ADAR and DICER1. ADAR also reduces PKR (protein kinase, dsRNA activated (*EIF2AK2*)) stress-related responses by editing dsRNA. ADAR p150 and PKR expression is stimulated by interferon. Decreased ADAR activity or increased production of dsRNA promotes translation of other ISG, leading to amplification of interferon responses through the dsRNA sensor MDA5 (encoded by *IFIH1*) that promotes the assembly of MAVS filaments on the mitochondrial (mt) surface. Loss of ADAR function is causal for Aicardi-Goutières Syndrome, a disease where the persistent activation of the interferon system is driven by endogenous dsRNA formed in part from Alu inverted repeats. Loss of DICER1 function in age-related macular degeneration leads to accumulation of Alumers, loss of mt integrity, release of mt nucleic acids, inflammasome activation and cell death

Not all Alumers (fragments from a single Alu element) form dsRNA editing substrates (Table 1). Additional counter measures to destroy Alumers include miRNAs produced by the DICER1 containing microRNA processor complex^65^. ADAR and DICER1 form a heterodimer via a protein:protein interaction and are thereby targeted to the same transcripts^86^. Overall, ADAR increases the efficiency of miRNA production. During stress, editing of pri-miRNA substrates by p150 increases the production of mature miRNA by DICER1^59^ (Fig. 5), restoring the RNA-based suppression of ISG protein production. By turning on RNA-world controls^87^, p150 switches off protein-based immunity.

DICER1 is associated with different diseases than ADAR. Loss of DICER1 function produces ARMD^88^. AMRD is characterized by a high level of cytoplasmic Alumers that induce the release of DNA from mitochondria^89^. The Alumers may sponge up SRP9/14, causing mis-targeting of proteins to mitochondria, leading to a loss of mitochondrial integrity and leakage of their contents into the cytoplasm^90^. Mitochondrial nucleic acids are sensed by cGAS (MB21D1) and by ZBP1 in epithelium^91^. Both proteins signal cooperatively through the STING-TBK1-IRF3 pathway to induce interferon-beta release and NLRP3 inflammasome activation, initiating the FAS/FASL-dependent cell death of retinal pigmented epithelium seen in ARMD^89,92,93^. Activation of ZBP1 could also be by Z-RNA as the best Z-element, gcgcgtacacac is from helix 28 of the mitochondrially encoded 12S RNA.

ADAR and DICER1 may jointly induce other pathologies. For example, GAC triplet repeats can form hairpins with noncanonical basepairs, either by DNA slippage or RNA foldback. Binding of Zα to these structures would also localize DICER1. The small RNAs made by DICER1 from hairpin substrates would induce a dominant negative disease phenotype by interfering with transcripts from both normal and mutant copies of the gene^94^. The outcome does not depend on the editing by ADAR. Targeting of other enzymatic machinery to Z-elements marking actively transcribed domains can generate many different outcomes.

## Roles for Zα independent of DNA

There are conditions where Zα may only target RNA. Stress granules form when dsRNA activates the kinase PKR^95^ and are more prone to form in ADAR-deficient cells due to the accumulation of dsRNA^44^. They are stabilized by dsRNA tangles caused by *trans*-RNA interactions between repetitive elements such as Alu^96^. Such tangles, trapped by topology, are capable of Z-formation^97^. Indeed, many Zα domain proteins localize to stress granules^98^. ADAR may not only prevent stress granule formation, but also may help resolve them by editing and destabilizing tangles.

## The known unknowns

The structure and function of Z-DNA has and will continue to generate many surprises. A world based on structural motifs enables fine-tuning of many phenotypes, with outcomes fashioned both by the length and positioning of shape elements within a genome. The regulated expression of proteins like ADAR p150 scales innate immune responses adaptively by changing the way transcripts are produced and processed. Challenges to further understanding Z-biology remain, both small and big. One task is to identify Zα family members with more divergent sequences to facilitate discovery of other Z-scaled outcomes. One question is whether Z-binding proteins exist that are sequence-specific. They may not. Evolution by shape can proceed faster using nucleotide variation to time when and where a structure forms. There is no need to tailor the proteins that bind it. The process can be accelerated by exploiting retroelements to spread shape motifs throughout a genome. Another task is to structurally confirm and define the interactions between Zα and noncanonical conformations like single-stranded Z-turns, (GU)_*n*_ dsRNA stems, triplet repeats and quadruplex forming sequences. Many sequences can form more than one shape. Competition for each alternative by structure-specific proteins will enable different outcomes. Also of great importance are functional studies to examine how variations in positioning and length of Z-forming segments alter responsiveness to environmental perturbations. Existing in vitro and in vivo assays of immune responses and tumor metabolism are suitable for such purposes. When combined, these approaches will provide insight into how genomes encode information by both shape and sequence to generate selectable phenotypic plasticity^99^. Conformations like Z-DNA are of special interest as they dynamically modify the readout of sequence information from the genome. By altering the location and timing of RNA processing events, they enable a range of rapid responses to environmental stress.

## Conclusion

This review captures roles for the Z-conformation in RNA- and protein-based immunity and describes parts played by the Zα domain in RNA-mediated diseases such as Aicardi-Goutières Syndrome and ARMD. A related theme focuses on the importance of Z-formation to the defense of primate genomes against the hordes of Alu invaders. During the many skirmishes, both RNA editing and RNA interference became weaponized with Z-formation likely supplying precision targeting coordinates, enabling the enzymes to curtail further attacks. The system evolved to regulate protein-centric innate immune responses against more sophisticated invaders, like viruses, which counter-attacked by perfecting their own Zα proteins. Another theme explores the many different ways in which Z-formation alters the readout of genomic information over relatively short time-periods. The change is not as fast as observed with post-translational modifications, such as phosphorylation or ubiquitination. The time scale is better suited to feedback mechanisms where alternative transcripts from a single gene calibrate responses by encoding contrary outcomes. This mode of genetic regulation is likely to vary between individuals and segregate with differences in disease risk. Long Z-DNA containing genes enriched for disease-causing mutations are one place to look for such associations.

## Supplementary information

Supplementary Information

Supplementary Data 1

Supplementary Data 2

Supplementary Data 3

Description of Additional Supplementary Files
