# Supplementary material for: Z-DNA and Z-RNA in human disease
Source: Commun Biol. 2019 Jan 7;2:7. doi: 10.1038/s42003-018-0237-x (PMC6323056; doi:10.1038/s42003-018-0237-x)
Supplement: Supplementary file 1 — Supplementary Information [file 42003_2018_237_MOESM1_ESM.docx]

**Supplemental Methods**

The hg19 (February 2009) build was scored for Z-DNA formation using the ZHUNT3 program developed by Dr. Shing Ho [^1^](#_ENREF_1)^,^[^2^](#_ENREF_2). The code was modified so that positions scored by ZHUNT3 corresponded to those in the genome release and to reformat the output to include both the sequence scored and the anti-syn assignment. The Linux utility awk was used to extract lines with Z-scores >250. Locations of repeat elements identified with RepeatMasker were obtained from the UCSC genome web-browser Table Bowser (<http://genome.ucsc.edu/cgi-bin/hgGateway>). The output is available at <https://datadryad.org/> using the DOI identifier for this publication.

The list of sites of adenosine to inosine editing was obtained from the RADAR Database Version 2.0 (http://rnaedit.com/). The BioConductor Packages Biostrings, IRanges, GenomicRanges, TxDb.Hsapiens, org.Hs.eg.db and UCSC.hg19.knownGene along with the R packages data.table stringr, reshape2 and dplyr were used in custom scripts written in the R language to annotate genes and overlap Z-DNA forming segments, repeats, genes and editing sites by chromosome. Figure 4 was generated using ggplot2, grid and gridExtra libraries. Alu sequences were obtained from <http://www.repeatmasker.org/>[^3^](#_ENREF_3), scored using ZHUNT3 and R Scripts used to generate Table 1. Scripts are available on request.

Gene enrichment long Z-DNA forming segment (Z-score >10,000) was performed using DAVID (<https://david.ncifcrf.gov/summary.jsp>). Disease gene annotation was obtained from uniprot.org([https://www.uniprot.org/uniprot/?query=reviewed:yes%20keyword:%22Disease%20mutation%20[KW-0225]%22](https://www.uniprot.org/uniprot/?query=reviewed:yes%20keyword:%22Disease%20mutation%20%5bKW-0225%5d%22)) and disease pathway enrichment performed using Enrichr (<http://amp.pharm.mssm.edu/Enrichr/>). RNA folds were determined using the RNAfold webserver (<http://rna.tbi.univie.ac.at//cgi-bin/RNAWebSuite/RNAfold.cgi>) and figures generated with VaRNA (<http://varna.lri.fr/>) [^4^](#_ENREF_4) and Weblogo(https://weblogo.berkeley.edu/logo.cgi)[^5^](#_ENREF_5). Crystal structures were obtained from the Protein DataBank (<https://www.rcsb.org/>) and figures prepared using the NGL Viewer (<http://proteinformatics.charite.de/ngl/html/ngl.html>).

**Supplemental References**

1. Champ, P.C., Maurice, S., Vargason, J.M., Camp, T. & Ho, P.S. Distributions of Z-DNA and nuclear factor I in human chromosome 22: a model for coupled transcriptional regulation. *Nucleic acids research* **32**, 6501-10 (2004).

2. Ho, P.S., Ellison, M.J., Quigley, G.J. & Rich, A. A computer aided thermodynamic approach for predicting the formation of Z-DNA in naturally occurring sequences. *The EMBO journal* **5**, 2737-44 (1986).

3. Smit, A.F.A., Hubley, R. & Green, P. RepeatMasker Open-4.0. 2013-2015. (2015).

4. Darty, K., Denise, A. & Ponty, Y. VARNA: Interactive drawing and editing of the RNA secondary structure. *Bioinformatics* **25**, 1974-5 (2009).

5. Crooks, G.E., Hon, G., Chandonia, J.M. & Brenner, S.E. WebLogo: a sequence logo generator. *Genome research* **14**, 1188-90 (2004).
