# Supplementary material for: Z-DNA and Z-RNA in human disease
Source: Commun Biol. 2019 Jan 7;2:7. doi: 10.1038/s42003-018-0237-x (PMC6323056; doi:10.1038/s42003-018-0237-x)
Supplement: Supplementary file 5 — Description of Additional Supplementary Files [file 42003_2018_237_MOESM5_ESM.docx]

**Description of Additional Supplementary Files**

**File Name**: Supplementary Data 1

**Description**: Coordinates for regions in hg19 (February 2009) human genome sequence release with Z-scores >250

**File Name**: Supplementary Data 2

**Description**: Coordinates for regions in hg19 (February 2009) human genome sequence release with Z-scores >10,000

**File Name**: Supplementary Data 3

**Description**: Sheet 1: Key Sheet 2: All Genes with long Z-DNA segment Sheet 3: DAVID Enrichment Sheet 4: Genes with long Z-DNA segment in the set enriched for disease-associated mutation Sheet 5: Disease Genes - Reactome Pathway Enrichment Sheet 6: Disease Genes - KEGG Pathway Enrichment Sheet 7: Disease Genes - Panther Pathway Enrichment Sheet 8: Disease Genes – NCI-Nature Pathway Enrichment Sheet 9: Disease Genes - WikiPathways Pathway Enrichment
